# The impact of home learning on myopia progression in primary school students during the COVID-19 pandemic

**DOI:** 10.1097/MD.0000000000047450

**Published:** 2026-01-30

**Authors:** Hao Luo, Dingyu Zhao, Juan Li

**Affiliations:** a School of Sports and Human Sciences, Beijing Sport University, Beijing, China; b School of Physical Education, Shanxi University, Taiyuan City, Shanxi Province, China; c College of Physical Education and Health, Guangxi University of Nationalities, Nanning City, Guangxi Province, China.

**Keywords:** COVID-19 pandemic, home learning, myopia progression, primary school students

## Abstract

This study aims to investigate the impact of home learning on myopia progression in primary school students during the COVID-19 pandemic and analyze associated risk factors. A visual acuity examination and a non-cycloplegic autorefraction were conducted on the subjects before the outbreak of COVID-19 and after classroom learning resumption, and a questionnaire survey was performed concerning visual behavior, physical activity, sleep duration, among others. The multiple linear regression analysis was utilized to analyze risk factors associated with myopia progression in primary school students. The total prevalence rates of myopia in primary school students before the COVID-19 pandemic and after classroom learning resumption were 17.4% and 42.1%, respectively (*P* < .001), and the mean spherical equivalent (SE) values were −0.24D and −0.85D respectively. The prevalence of myopia in 5th and 6th graders was significantly higher than that in 1st through 4th graders and the SE values were considerably lower than those in 1st through 4th graders. The multiple linear stepwise regression analysis showed that myopia progression was significantly associated with screen time and the number of parents with myopia. Among them, parental myopia alone accounted for 16% of the variation in SE (ΔSE), and parental myopia and screen time in combination could account for 29% of ΔSE. Home learning during the COVID-19 pandemic accelerates myopia progression in children, so efforts should be made to optimize the educational program for home learning, particularly to diminish daily screen time and meanwhile increase outdoor time.

## 1. Introduction

The growing prevalence of myopia around the globe over the past 3 decades^[1,2]^ has drawn wide attention and concern. The World Health Organization estimates that by 2050, with increasing urbanization, half of the world’s population will be affected by myopia, with high myopia prevalence of up to 10%.^[3]^ Statistics from the National Health Commission of the People’s Republic of China indicated that in 2020 the overall myopia rate among Chinese children and adolescents reached 52.7%, 2.5% up compared to that in 2019. The increase is attributable to decrease in outdoor time due to the COVID-19 epidemic.^[4]^ Myopia presents a major risk of severe ocular complications^[5]^ and significantly affects health and quality of life across the lifespan, and therefore an exploration of the risk factors associated with myopia progression is of great clinical value and social significance to myopia prevention and control. Myopia onset and progression are closely linked to multidimensional risk factors, and internal factors include age, gender, and genetic factors,^[6]^ among others. Myopia mostly starts around school-age, so the primary school period plays a crucial role in myopia prevention and control. It is noteworthy that the increase in digital screen time is an important external risk factor associated with myopia.^[7]^ In addition, environmental factors are associated with myopia progression, including near-work time,^[8]^ sleep duration,^[9]^ and electronic screen type.^[10]^ Outdoor time is considered to be the most prominent environmental factor for myopia control.^[11,12]^ Due to the COVID-19 epidemic, Chinese students were obliged to have classes online in the second half of the academic year 2019 to 2020, and the change in learning mode substantially increased digital screen time and near-work time and reduced outdoor time in schoolchildren, thus posing serious problems to eye health. This study involved 2 tests, a preliminary test conducted in November 2019, including a visual acuity examination and a refractive error test, and a follow-up test performed after classroom learning resumption in June 2020, including the same examination and test with a stratified sampling method. The subjects were 720 students from 4 primary schools in Shanxi Province. Furthermore, a questionnaire survey was also administered to the subjects concerning visual behavior, physical activity, sleep duration, and other relevant factors during home learning to analyze myopia progression and risk factors in primary school students during the particular period.

## 2. Materials and methods

### 2.1. General data

This study was approved by the Ethics Committee of Guangxi University of Nationalities (2020-1208-No.48). Based on the visual acuity examination and refractive error test conducted on primary school students in Shanxi Province in November 2019, 4 of the primary schools were randomly selected for the follow-up test in June 2020. A total of 720 students, who were selected by stratified sampling, underwent all tests and completed the questionnaire. Among them, 180 students were selected from each school, with 30 students (male: 15, female: 15) in each grade. This study adopted a pretest–posttest repeated-measures cohort design, in which the same group of primary school students underwent vision examinations before the COVID-19 outbreak and again after the resumption of classroom learning. Exclusion criteria: students with known ocular diseases other than refractive errors (e.g., amblyopia, strabismus, congenital eye abnormalities); students with systemic diseases affecting vision; students who had undergone any ophthalmic surgery; students who failed to complete both baseline and follow-up examinations; and students whose questionnaire data were incomplete or invalid. No loss to follow-up occurred, and no sample replacement was required.

### 2.2. Methods

Based on the vision test questionnaire of the National Students Physical Health Test, considering the study and lifestyle change during the Covid-19, the new version of the questionnaire is finally determined through group discussion. The questionnaire is contacted when the students were given the vision test after the epidemic and were completed by the students under the guidance of their parents. It should be noted that in the follow-up test, the testers were admitted to school after rigorous epidemic prevention inspection. This study has been reviewed and approved by the medical ethics committee of Shanxi University and the experimental process conforms to the ethical principles laid down by the Helsinki Declaration of the World Medical Association. All patients and their families have been informed of this study and signed the informed consent form. (A non-cycloplegic autorefraction is always conducted in general investigation of myopia prevalence in children in China).

### 2.3. Indicators

Such data as height, weight, and body mass index (BMI) were recorded in the preliminary test. In both tests, all subjects underwent visual acuity examination and non-cycloplegic autorefraction. The schools had implemented lockdown measures due to the pandemic, so it was impossible to conduct a non-cycloplegic autorefraction on the subjects and ideal conditions for the experiment failed to occur. The Topcon RM8000 Auto Refractometer (Topcon Co., Tokyo, Japan) was utilized for at least 3 measurements for each eye in the non-cycloplegic autorefraction. If the difference between any 2 measurements in diopter of spherical power was equal to or >0.50D, an extra measurement would be made and the mean of 3 reliable measurements was employed for subsequent analysis.

Information regarding the subjects’ visual behavior, lifestyle, etc during the pandemic was collected through a questionnaire with parental help. The questionnaire mainly covered screen time on weekdays and weekends, off-screen near-work time, sleep duration, indoor time, parental myopia history, among others. Screen time refers to the aggregate amount of time spent on mobile phones, televisions, tablet computers, and other electronic devices per day; off-screen near-work time refers to the aggregate amount of time spent doing homework, reading, drawing, and the like; the questionnaire only covered indoor activities since citizens were prohibited from unnecessary trips during the outbreak of COVID-19; sleep duration included nighttime sleep time and afternoon nap time.

### 2.4. Relevant definitions

To reduce the false positive rate of myopia, myopia was defined as spherical equivalent (SE) < −0.50D (refractive error) in either eye with visual acuity (uncorrected visual acuity) < 5.0.^[13]^ The variation in SE (ΔSE) in the right eye in the 2 tests were incorporated into risk factors of myopia progression during the pandemic. Screen time was obtained by the following formula: screen time = (weekday hours × 5 + weekend hours × 2)/7; likewise, off-screen near-work time, sleep duration, and indoor time were obtained by this formula as well.^[14]^

### 2.5. Statistical analysis

The descriptive statistical method was utilized for general data collection; a chi-square test was used for comparative analysis of the prevalence of myopia in male and female students and that before and after the pandemic; the variance analysis of repeated measurement was employed for comparative analysis of the SE values in the 2 tests; the 1-factor variance analysis was performed to compare SE and ΔSE in different grades and the multiple comparative analysis was conducted according to the results; an independent samples *t*-test was performed to compare SE values in male and female students in the 2 tests; a refractive error test was conducted on each subject to calculate ΔSE in the right eye in both tests. The multiple linear stepwise regression analysis was used to analyze the risk factors associated with myopia progression and to build a multiple linear regression model. The 1-factor variance analysis and multiple comparative analysis were utilized to compare the ΔSE in children with different numbers of parental myopia. Data analysis was performed with SPSS 24.0 software (IBM Corp., Armonk, NY, USA) and the difference with *P* < .05 was statistically significant.

## 3. Results

### 3.1. Demographic information

A total of 720 (female:360, male:360) subjects from 4 primary schools participated in the study. The mean age, height, weight and BMI were 9.0 ± 4.4 years, 136.8 ± 12.0 cm, 33.0 ± 10.9 kg and 17.2 ± 3.4 kg/m^2^ respectively. The demographic information of the subjects of different grades is detailed in Table 1.

**Table 1:** Basic information of subjects (2019.11).

Parameters	Overall (720)	Grade 1	Grade 2	Grade 3	Grade 4	Grade 5	Grade 6
Number of subjects (male/female)	360/360	60/60	60/60	60/60	60/60	60/60	60/60
Age (yr)	9.0 (4.4)	6.4 (0.3)	7.5 (0.2)	8.4 (0.2)	9.6 (0.3)	10.4 (0.2)	11.5 (0.5)
Height (cm)	136.8 (12.0)	121.1 (4.6)	127.4 (4.2)	132.4 (4.7)	138.7 (5.0)	144.8 (4.8)	152.6 (7.0)
Body weight (kg)	33.0 (10.9)	22.8 (3.3)	25.5 (4.3)	29.1 (6.2)	33.4 (7.0)	39.0 (8.4)	45.3 (11.2)
BMI (kg/m^2^)	17.2 (3.4)	15.5 (1.6)	15.7 (2.1)	16.6 (2.9)	17.3 (3.0)	18.5 (3.6)	19.3 (4.1)

The values are presented as the means (standard deviations).

BMI = body mass index.

### 3.2. Number of patients with myopia and prevalence rates

**Figure 1.**
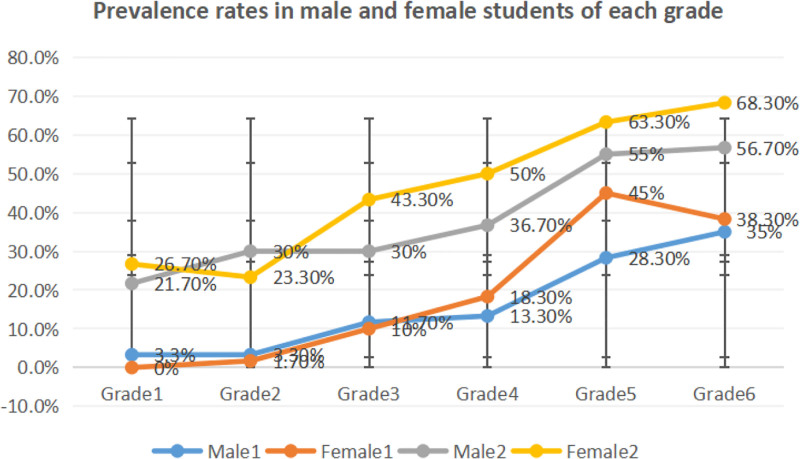
Prevalence rates in male and female students of each grade. Male 1 and female 1 indicate corresponding prevalence rates in the preliminary test and male 2 and female 2 in the follow-up test.

In both tests, the number of patients with myopia and prevalence rate increase with grade level, particularly in 5th and 6th graders, as is shown in Table 2 and Figure 1. The chi-square test results showed no significant difference (χ^2^ = 1.171, *P* > .05) in prevalence rate between male and female students in the preliminary test; the prevalence rate in female students was significantly higher than that in male students (χ^2^ = 4.154, *P* < .05) in the follow-up test. Prevalence rates in both male and female students in the follow-up test were significantly higher than those in the preliminary test (χ^2^ = 46.143, χ^2^ = 59.702, *P* < .001).

**Table 2:** Number of patients with myopia and comparison.

	2019 (persons)	2020 (persons)	
Grade 1	2	29	
Grade 2	3	32	
Grade 3	13	44	
Grade 4	19	52	
Grade 5	44	71	
Grade 6	44	75	
Male	57	138	χ^2^ = 46.143**
Female	68	165	χ^2^ = 59.702**
	χ^2^ = 1.171	χ^2^ = 4.154*	

* Indicates *P* < .05.

** Indicates *P* < .001.

### 3.3. SE values

**Figure 2.**
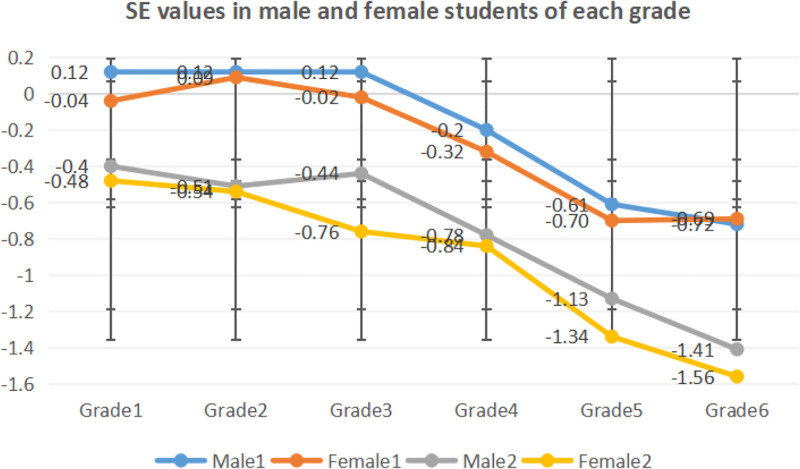
SE values in male and female students of each grade. Male 1 and female 1 indicate the SE values in the preliminary test and male 2 and female 2 in the follow-up test. SE= spherical equivalent.

Comparison of the SE values in the 2 tests using the variance analysis of repeated measurement revealed that the outcomes of the preliminary test were remarkably lower than those of the follow-up test in both male and female students of different grades. Comparison of the SE and ΔSE values in different grades using 1-factor variance analysis and multiple comparative analysis indicated the following: the SE values in 5th and 6th graders were significantly lower than those in 1st through 4th graders (*P* < .01), with 4th graders significantly lower than second graders in 2019; in 2020, the SE values in 5th and 6th graders were considerably lower than those in 1st through 4th graders (*P* < .01), with 4th graders remarkably lower than 1st graders (*P* < .05); the ΔSE values in the 6th graders were significantly higher than those in 1st graders (*P* < .01), as is shown in Table 3. The SE values in male and female students through independent samples *t*-test indicated that difference in gender only showed in third graders in the follow-up test (*P* < .05), as is illustrated in Figure 2.

**Table 3:** SE and ΔSE values of 2 tests.

	Grade 1	Grade 2	Grade 3	Grade 4	Grade 5	Grade 6	Overall
2019 (D)	0.04 (0.74)	0.11 (0.73)	0.05 (0.84)	−0.26 (0.98)	−0.66 (1.03)	−0.70 (0.99)	−0.24 (0.95)
2020 (D)	−0.44 (0.75)*	−0.52 (0.78)*	−0.60 (0.86)*	−0.81 (1.04)*	−1.24 (1.20)*	−1.49 (1.30)*	−0.85 (1.08)*
ΔSE	−0.48 (0.86)	−0.64 (0.92)	−0.64 (0.89)	−0.56 (0.75)	−0.57 (0.77)	−0.79 (1.04)†	−0.61 (0.88)

The values are presented as the means (standard deviations).

SE = spherical equivalent.

* Indicates comparison between the 2 tests, *P* < .01.

† Suggests comparison between grade 6 and grade 1 in terms of ΔSE, *P* < .01.

### 3.4. Analysis of risk factors

During the COVID-19 epidemic, daily screen time and off-screen near-work time increase with grade level whereas outdoor time and sleep duration decline with grade level, as is shown in Table 4. A multiple linear stepwise regression analysis was conducted with the above parameters and the subjects’ gender, BMI and age as independent variables and ΔSE as the dependent variable, and the regression models constructed are shown in Table 5. Model 1 incorporated a mere independent variable (the number of parents with myopia), which alone accounted for 16% of the ΔSE, and model 2 included 2 independent variables (the number of parents with myopia and screen time), which in combination accounted for 29% of ΔSE, and other parameters were not incorporated into the regression models. The outcomes of the 1-factor variance analysis suggest that the values of ΔSE in schoolchildren with both parents affected by myopia were significantly higher than those with zero parent affected by myopia (*P* = .001) and those with 1 parent affected by myopia (*P* = .030; see Table 6).

**Table 4:** Statistics of risk factors for home learning during the epidemic.

Independent variable	Grade 1	Grade 2	Grade 3	Grade 4	Grade 5	Grade 6
Screen time (min)	210.5 (96.8)	216.2 (100.0)	248.6 (106.4)	251.5 (100.1)	273.2 (95.4)	278.9 (88.7)
Off-screen near-work time (min)	186.3 (56.2)	191.0 (91.2)	211.7 (73.4)	211.6 (51.8)	230.3 (86.4)	235.9 (56.6)
Indoor time (min)	62.3 (48.5)	69.3 (56.8)	58.2 (57.0)	58.6 (35.2)	57.7 (52.5)	53.6 (54.7)
Sleep duration (min)	546.7 (50.5)	530.3 (48.6)	526.8 (41.7)	529.0 (33.6)	529.1 (33.9)	522.1 (42.0)
Number of parents with myopia (persons)						
0	51 (42.5%)	52 (43.3%)	59 (49.2%)	65 (54.2%)	66 (55.0%)	60 (50.0%)
1	42 (35.0%)	53 (44.2%)	40 (33.3%)	35 (29.2%)	34 (28.3%)	38 (31.7%)
2	27 (22.5%)	15 (12.5%)	21 (17.5%)	20 (16.6%)	20 (16.7%)	22 (18.3%)

The values are presented as means (standard deviations) or values (percentages).

**Table 5:** Multiple linear stepwise regression analysis model of ΔSE and independent variables.

Model (standardization coefficient)	*R* ^2^	*F*	*P*
ΔSE = −0.127 × Number of parents with myopia	0.152	63.811	<.001
ΔSE = −0.133 × Number of parents with myopia − 0.124 × screen time	0.291	146.116	<.001

SE = spherical equivalent.

**Table 6:** Multiple comparison analysis of ΔSE in children with different numbers of parents with myopia.

	0	1	2
Number of subjects (%)	353 (49.0%)	242 (33.6%)	125 (17.4%)
ΔSE value	−0.52 (0.84)	−0.63 (0.92)	−0.85 (0.85)*,†

SE = spherical equivalent.

* Indicates a significant difference between children with both parents affected by myopia and those with 1 parent with myopia, *P* < .05.

† Indicates a significant difference between children with both parents suffering from myopia and those with no parent affected by myopia, *P* < .01.

## 4. Discussion

The outcomes of the study indicate that in the preliminary test, prevalence rates in 1st through 6th graders were 1.7%, 2.5%, 10.8%, 15.8%, 36.7%, and 36.7% respectively, with an abrupt increase in 4th and 5th graders, probably due to the heavy study load before the Junior Middle School Entrance Examination. According to statistics from Hong Kong, the figures are 13.3%, 30%, 42.7%, 38.1%, 53.6%, and 54.7% respectively,^[15]^ much higher than Shanxi. In Beijing, the prevalence rates of myopia in children aged 7 to 11 are 11.7%, 14.6%, 31%, 37% and 46.7% respectively,^[16]^ close to those in Shanxi. In a nutshell, the severity of myopia found in Shanxi schoolchildren has become a serious public health concern.

Research indicates that the prevalence of myopia in both male and female students increases with the grade level, and that there is a positive correlation between age and the prevalence of myopia. This is probably due to increased academic burden, reduced outdoor time, and increased time spent on study.^[17]^ Primary school students with myopia run a higher risk of developing high myopia, which might lead to glaucoma, cataract, retinal detachment and other ocular diseases.^[18,19]^ Furthermore, female students have a higher prevalence rate than male students in both tests, reaching the level of significance in the follow-up test (*P* < .05), with the SE values lower than male students in both tests (see Fig. 2). A majority of studies show that for the same age group, girls have higher prevalence rate and severity than boys,^[20,21]^ which has been confirmed by the outcomes of this study. The potential reason for this gender difference lies probably in the fact that children in growth spurt, which occurs earlier in girls than in boys,^[22]^ undergo early peak SE and higher axial length growth speed and earlier myopia onset. Primary school students who develop fast run a higher risk of developing myopia. Hence, visual health in low graders should be given due attention to and the deferral prevalence age is of vital importance to the alleviation of the prevalence and progression of myopia.

Studies show that screen time and the number of parents with myopia are the key factors associated with myopia progression in home learning. During the COVID-19 pandemic, digital devices are the only means by which students have classes, so their daily exposure time to screen is prolonged to a large extent. A nationwide cross-sectional survey of 1,31,859 students revealed that the average daily screen time was 114 minutes.^[23]^ Through logistic regression analysis, Ma found that the SE values in the right eyes were relatively high in children with COVID-19 (−0.93 (0.65) vs −0.33 (0.47) D; *P* < .001) and that the SE values were significantly correlated to axial length (*P* = .028), online learning (*P* = .02) and screen time.^[24]^ As can be seen in Table 4, the average amount of daily screen time in primary school students during the pandemic roared to 246 minutes, considerably longer than that during the period of normal classroom learning. An American study has found that excessive screen use (4 hours/d) is associated with low psychological well-being, including reduced curiosity, poorer self-control and concentration, emotional instability, and inability to accomplish tasks.^[25]^ It can be noted that prolonged use of screen should be given adequate attention to considering its hazards to the body and the mind.

Once digital learning becomes prerequisite for regular study rather than being complementary, then even when the pandemic terminates, indoor time and screen time in school-age children will increase by osmosis. By comparing the rates of myopia in children during 2015 to 2019 and those in 1,23,535 children during the COVID-19 pandemic, Wang discovered that home isolation resulted in a substantial change toward myopia of approximately −0.3D, among which the amounts of change in 6-year-olds, 7-year-olds, and 8-year-olds were −0.32D, −0.28D and −0.29D respectively and that the amounts of change in prevalence rate of myopia were 21.5% vs 5.7%, 26.2% vs 16.2%, 37.2% vs 27.7% in children aged 6, 7, and 8 respectively.^[26]^ Moreover, a number of schools require students’ parents to provide their children with screen-based devices with a view to implementing home learning schemes. While digital technology has indisputably brought much convenience to people’s life, what is of paramount importance is that parents should help their children to foster a healthy relationship with digital devices. Screen time should be maximized for academic experience rather than extraneous content such as entertainment; screen time should be limited and frequent breaks are necessary during screen work and other near-work; increased physical activities are expected. In addition, parents themselves should set a good example to their children by reducing the amount of time spent on digital devices, increasing outdoor time with kids, and engaging them in offline games and non-digital indoor activities such as housework and handicraft art.^[27,28]^

Myopia results from both genetic and environmental factors. Despite the substantial influence of environmental factors on myopia progression, the number of parents with myopia and the severity of myopia, according to Tedja et al,^[29]^ are both factors associated with myopia progression,^[30]^ and the former, according to this study, is the leading influencing factor of myopia progression in children. As is presented in Table 6, significant difference exists in the amount of change of SE values between children with both parents affected by myopia and those with 0 or 1 parent affected by myopia. While there is no considerable difference in the variation of SE (ΔSE) between children with 1 parent with myopia and those with 0 parent with myopia, the value of ΔSE in children with 1 parent with myopia is higher than that in children with 0 parent with myopia. Twenty-five genetic loci (MYP1-MYP 25) have been confirmed to be associated with myopia onset.^[31,32]^ With the rapid development of molecular biotechnology, genetic research on myopia is still continuing. Given the huge number of myopia cases in China in recent years, there is a need to conduct a large-scale genetic survey on myopia and to investigate the mutations and regulatory mechanisms of disease genes, with a view to providing a powerful basis for myopia prevention and treatment.

In conclusion, the prevalence of myopia in primary school students is relatively high and increases with age. Home learning during the COVID-19 pandemic accelerates myopia onset and progression in children, which are closely related to daily screen time and parental myopic tendency. Against the backdrop of prolonged epidemic, all mankind should improve prevention and self-protection to diminish the spread of virus, and meanwhile optimize the educational program for home learning.

The limitations with this paper include 2 aspects. First, there are chances of overestimating the degree of myopia. With a large sample size, the refractive error test was conducted under quick and convenient non-cycloplegic conditions on account of time costs and experimental conditions, which might lead to degree of myopia overestimation. A study on myopia in children aged 5 to 6 revealed SE values in cycloplegic refraction were significantly more hyperopic than those in non-cycloplegic refraction by mean differences of (1.48 ± 1.31) diopters. Another study on myopia, in adults aged 30 to 83, also revealed there was an obvious hyperopia drift of SE after cycloplegia, from (−0.37 ± 1.22) D before test to (0.13 ± 1.11) D after test, and that it decreased with age. The 2 studies reached similar conclusions.^[33,34]^ Second, there are also chances of inaccuracy in the information given by the respondents. Questionnaire filling by low graders, who have a poor cognitive level, called for parental help, so there might be some bias in the relevant data compared to those directly from high graders. In future studies, the cycloplegic refraction test can be added and sample sizes should be enlarged, thus making the results more reliable.

## Author contributions

**Conceptualization:** Hao Luo, Dingyu Zhao, Juan Li.

**Data curation:** Hao Luo, Dingyu Zhao, Juan Li.

**Formal analysis:** Hao Luo, Dingyu Zhao, Juan Li.

**Funding acquisition:** Hao Luo, Dingyu Zhao, Juan Li.

**Investigation:** Hao Luo, Dingyu Zhao, Juan Li.

**Writing—original draft:** Hao Luo, Juan Li.

**Writing—review & editing:** Hao Luo, Juan Li.
